# Identification of targets of monoclonal antibodies that inhibit adhesion and growth in *Mycoplasma mycoides* subspecies *mycoides*

**DOI:** 10.1016/j.vetimm.2018.09.002

**Published:** 2018-10

**Authors:** Racheal Aye, Yenehiwot Berhanu Weldearegay, Harrison Osundwa Lutta, Francis Chuma, Andreas Pich, Joerg Jores, Jochen Meens, Jan Naessens

**Affiliations:** a Department of Biosciences, International Livestock Research Institute, P.O. Box 30709-00100, Nairobi, Kenya; b Department of Infectious Diseases, Institute for Microbiology, University of Veterinary Medicine Hannover, Hannover, Germany; c Kenya Agricultural and Livestock Research Organization-Biotechnology Research Institute, P.O. Box 14733-00800, Nairobi, Kenya; d Core Unit Proteomics, Hannover Medical School, Hannover, Germany

**Keywords:** *Mycoplasma mycoides* subspecies *mycoides*, Monoclonal antibodies, Adhesion inhibition, Growth inhibition

## Abstract

*Mycoplasma mycoides* subspecies *mycoides* (*Mmm*) adhesion is tissue and host specific. Inhibition of adhesion will prevent *Mmm* from binding to lung cells and hence prevent colonization and disease. The aim of this study was to develop a panel of *Mmm* monoclonal antibodies against *Mmm* and use these antibodies to investigate their inhibitory effect on the adherence of *Mmm* to bovine lung epithelial cells (BoLEC), and to further identify an antigen to any of the inhibitory antibodies. Thirteen anti-*Mycoplasma mycoides* subsp. *mycoides* (AMMY) monoclonal antibodies (mAbs) inhibited adhesion by at least 30% and ten of the mAbs bound to multiple bands on Western blots suggesting that the antibodies bound to proteins of variable sizes. AMMY 10, a previously characterized *Mmm*- capsular polysaccharide (CPS) specific antibody, inhibited growth of *Mmm in vitro* and also caused agglutination of *Mmm* total cell lysate. AMMY 5, a 2-oxo acid dehydrogenase acyltransferase (Catalytic domain) (MSC_0267) specific antibody, was identified and polyclonal rabbit serum against recombinant MSC_0267 blocked adhesion of *Mmm* to BoLEC by 41%. Antigens recognized by these antibodies could be vaccine candidate(s) and should be subsequently tested for their ability to induce a protective immune response *in vivo*.

## Highlights

• A panel of anti-*Mmm* mAbs was produced and screened for host-pathogen inhibition.
• 13 mAbs inhibited adhesion of *Mmm* to host target cells.
• Anti-capsular polysaccharide inhibited growth and caused agglutination of *Mmm*.
• Anti-PDHC inhibited adherence of *Mmm* cells showing the possible role of glycolytic enzymes in host-pathogen interaction.
• One novel antigen that is a promising vaccine candidate against CBPP identified.

## 1 Introduction

Many mycoplasmas that infect livestock adhere to and colonize epithelial surfaces of various tissues in their hosts. Adhesion is thought to be tissue and host specific, and a prerequisite for colonization by pathogenic mycoplasmas (Barile and Rottem, 1993). The loss of adhesion by mutation results in loss of infectivity, and reversion to the cytoadhering phenotype is accompanied by requisition of infectivity and virulence (Razin and Jacobs, 1992; Baseman and Tully, 1997). Cytoadherence of mycoplasmas to host cells may result in damage by interference with membrane receptors or alteration of transport mechanisms of the host cell (Debey and Ross, 1994), release of cytotoxic metabolites (Pilo et al., 2007) or hydrolysis of host cell phospholipids by potent membrane-bound phospholipases present in many *Mycoplasma* species (Shibata et al., 1995). The cytoadherence mechanisms of many *Mycoplasma* species, including those of *M*. *pneumoniae* (Chaudhry et al., 2007), *M*. *genitalium* (Ueno et al., 2008), *M*. *suis* (Zhang et al., 2015), *M*. *hominis* (Brown et al., 2014), *M*. *hyopneumonia* (Burnett et al., 2006), *M*. *agalactiae* (Fleury et al., 2002) and *M*. *bovis* (Song et al., 2012) have been described. In these studies, adherence was shown to be a complex multifactorial process involving one or more adhesion molecules.

In our previous study, we established a cytoadherence assay that measured the interaction of *Mmm* with various host cells and showed that *Mmm* cytoadherence is tissue and host specific (Aye et al., 2015). In this study we further analyzed the adhesion of *Mycoplasma mycoides* subspecies *mycoides* (*Mmm*), the causative agent of contagious bovine pleuropneumonia (CBPP), an important disease of cattle in sub-Saharan Africa.

The aim of this study was to identify potential *Mmm* vaccine targets by developing a panel of monoclonal antibodies against *Mmm*, and use them to investigate their inhibitory effect on the adherence of *Mmm* to primary bovine lung epithelial cells (BoLEC), their capacity to inhibit *Mmm* growth *in vitro*, and to further identify an antigen to which any one of the inhibitory antibodies bound. Using an indirect flow cytometry assay, we identified 13 anti-*Mycoplasma mycoides* subspecies *mycoides* antibodies (AMMY 1–13) that inhibited adhesion of *Mmm* to BoLEC. Subsequent to this initial work, Schieck et al (2016) characterized AMMY 10 as an *Mmm*-specific capsular polysaccharide (CPS) antibody. We further identified *pdhC*, part of the pyruvate dehydrogenase (PDH) complex, as an antigen that bound to AMMY 5 and provided evidence of its possible involvement in the cytoadhesion. *Mycoplasma* molecules involved in the adhesion process, as well as molecules essential for growth or important biological functions are candidate vaccine targets.

## 2 Materials and methods

### 2.1 *Mmm*-specific monoclonal antibody production

Five 6–8 week old BALB/c mice were immunized intraperitoneally with 100 μg *Mmm* strain Afadé whole cell lysate in 100 μl PBS mixed with equal volumes of TitterMax adjuvant (Sigma) on days 0, 14 and 21. Seven days after the last immunization, antibody titers were measured by indirect enzyme-linked immunosorbent assay (ELISA-see below). Mice with highest antibody titers were given a booster dose of 100 μg *Mmm* lysate without adjuvant three days before aseptic spleen removal for fusion. Mice were used in accordance with the International Livestock Research Institute Institutional Care and Use Committee guidelines (IACUC Ref nr 2010.06)

Fusion was carried out as previously described in detail (Naessens et al., 1985) using X63Ag8.653 murine myeloma cells. The hybridomas were cultured in 96-well Costar tissue culture plates (Sigma) in the presence of thymocytes, hypoxanthine, aminopterin, and thymidine (Sigma) at 37 °C in 5% CO_2_ for 10–14 days.

Hybridomas were screened for antibody secretion by standard indirect ELISA. Briefly, Immunlon 2 HB plates (Dynatech Laboratories) were coated with 5 μg/ml *Mmm* strain Afadé whole cell lysate (100 μl/well) and incubated overnight at 4 °C. Non-specific binding sites were blocked with PBS containing 0.1% Tween 20 (PBST) and 5% skim milk for 1 h at room temperature. The plates were incubated with 100 μl hybridoma culture supernatants and then peroxidase-conjugated anti-mouse antibody (Sigma) for 1 h at room temperature. Plates were washed thrice with PBST after each incubation step. Reactions were visualized by adding 3,3’,5,5’ tetramethylbenzidine (TMB) substrate and the reaction stopped with 75 μl 2 *N* hydrochloric acid. Plates were read at 450 nm using a microplate ELISA reader. Positive hybridomas were cloned by limiting dilution before being scaled up for further testing.

### 2.2 Inhibition assay

#### 2.2.1 *Mycoplasma mycoides* subspecies *mycoides* culture

*Mmm* strains Afadé, Gladysdale and PG1 were cultured in 20 ml “pleuropneumonia –like organism” (PPLO) medium (Becton Dickinson, Park, USA) supplemented with 10% horse serum (Sigma, St Louis, USA), 0.9 g yeast extract/l, 0.5% glucose and 0.03% penicillin G at 37 °C for 48 h to a density of 10^8^ CFU/ml. Titration of *Mycoplasma* in the culture was carried out by the standard method of microtitration and color change and calculation of the titre by using the Spearman–Karber formula

(Litamoi et al., 1996) before centrifugation. The mycoplasmas were harvested by centrifugation at 6000 × *g* at 4 °C for 30 min, washed once in Dulbecco’s modified eagle′s medium (DMEM) without supplements and suspended in 10 ml of the same medium. Final titers were calculated using optical density at _OD_650 and readings plotted on a standard curve based on correspondence between _OD_650 and *Mmm* numbers as determined by TaqMan Real Time PCR.

#### 2.2.2 Cytoadherence inhibition assay

All ELISA-positive *Mmm*-specific monoclonal antibodies were tested for their ability to inhibit adhesion to BoLEC. Primary BoLEC were cultured using the protease digestion technique as described elsewhere (Thomas et al., 2003) and cultured in 24 well plates to a density of approximately 1.5 × 10^5^ cells per well.

*Mmm* strain Afadé specific monoclonal antibodies (AMMY) were diluted in PBS and pre-incubated with 200 μl of *Mmm* (approximately 1.5 × 10^8^ mycoplasmas) for 1 h at 37 ° C before adding them to the BoLEC. Experiments were performed three times in duplicate.

The cytoadherence assay and flow cytometry analysis were performed as previously described (Aye et al., 2015). The proportion of cells to which mycoplasmas had bound was calculated using Flow Jo (FLOWJO, 2014).

Percentage inhibition was calculated by the formula

$$\frac{\%\;+ve\;\;cells\;of\;\;sample\;\;without\;\;antibody\;-\;\%+ve\;cells\;\;of\;\;sample\;with\;\;the\;antibody}{\%\;+ve\;\;cells\;\;of\;the\;sample\;without\;antibody}\times \;100$$

Antibodies that inhibited adhesion were isotyped using the rapid ELISA mouse isotyping kit (ThermoFisher Scientific) according to the manufacturer’s instructions.

### 2.3 Antigen identification

#### 2.3.1 SDS-PAGE and Western blotting

*Mmm* strain Afadé was cultured as described above, a lysate prepared by ultra-sonication and the protein concentration determined by micro BCA (ThermoFisher Scientific) according to the manufacturer’s instructions.

Approximately 250 μg of lysate was diluted 1:2 in SDS sample buffer and incubated for 5 min at 95 °C, loaded on to a single comb discontinuous Tris/glycine SDS-PAGE mini-gel (12.5% polyacrylamide resolving gel; 3% acrylamide stacking gel) and subjected to electrophoresis as described previously (Laemmli, 1970). 5 μl of a prestained molecular weight marker (PageRuler™ Prestained Protein Ladder, Thermo Scientific) was loaded in a separate lane to permit determination of the apparent molecular weight of protein(s) of interest. Electrophoresis was performed using the Hoefer electrophoresis system (Serva Electrophoresis GmbH, Heildelberg, Germany) at 120 V for 2 h.

After SDS-PAGE, *Mmm* proteins were transferred onto nitrocellulose membrane (0.45 μm; Bio-Rad Laboratories, Inc., Hercules, CA) using the Hoefer electroblotting system (Serva Electrophoresis GMbH, Heildelberg, Germany) at 50 V for 1 h. Non-specific binding sites on the membrane were blocked using 5% BSA/PBS for 1 h at room temperature, washed thrice in PBST, air dried for 30 min at room temperature and cut into 4 mm strips before being incubated with the different AMMY mAbs (1:1000) for 1 h at room temperature, followed by washing and incubation with polyvalent goat anti- mouse antibody conjugated to HRP (1:2500; Sigma) for 1 h at room temperature. After a further 5 washes with PBST, bands were visualized by incubation with 30% diaminobenzidine (DAB) and 1% hydrogen peroxide in Tris-buffered saline until sufficient resolution was achieved. The reaction was stopped by washing the membrane with distilled water.

The specificity of AMMY mAbs for only members of a particular subspecies in the closely related *Mycoplasma mycoides* cluster was assessed. Lysates from four subspecies including *Mmm* (strains Afade, B237, B66 and T1/44), *M*. *mycoides* subspecies *capri* (strains 80/93, 83/90 and 153/90), *M*. *capri* subspecies *capripneumoniae* (strains Calf kid and 7714) and *M*. l*eachii* (strains FRD42 and 4146) were tested. The proteins were loaded onto a 15 well gel, and electrophoresis and Western blotting were performed as described above.

#### 2.3.2 2DE, immunoblotting, trypsin digest and mass spectrometry

Protein preparation and 2-dimensional electrophoresis (2DE) were performed as described by Jores et al. (2009). First dimension focusing was performed on 24 cm non-linear pH gradient (4–7 NL) strips (GE Healthcare, Germany) for 21 h (3 h at 150 V, 3 h at 300 V, 6 h at a 1000 V gradient, 3 h at an 8000 V gradient, and 6 h at 8000 V).

Separation in the second dimension was performed by SDS-PAGE on 12.5% polyacrylamide gels at 12 °C and 50 V for 2 h then at 15 V per gel overnight using the EttanTM DaltSix Electrophoresis System (GE Healthcare, Germany). All experiments were run in duplicate. Proteins on the first gel were placed in 400 ml fixative (45% methanol and 1% acetic acid in water) for 1 h at room temperature and visualized by staining with colloidal Coomassie blue stain as previously described (Weldearegay et al., 2016).

The proteins in the second gel were electro blotted onto a nitrocellulose membrane using a semi-dry Multiphor II (GE Healthcare) at 0.8 mA/cm^2^ for 1 h. Non-specific binding sites were blocked and antibody binding spots detected as described above. Immunoblots were developed using alkaline phosphatase-labeled goat anti-mouse antibody (Dianova, Hamburg, Germany, 1:2500 dilution) and 5-bromo-4-chloro-3- indolyphosphate combined with nitrotetrazolium blue chloride (BCIP/NBT; both Sigma, Germany).

Spots of interest were excised from the colloidal Coomassie blue-stained gel and subjected to standard in-gel de-staining, reduction, alkylation, trypsin digestion and peptide extraction as described elsewhere (Shevchenko et al., 2007), and then dried using a DNA Speedvac concentrator (Thermo Fisher Scientific) for mass spectrometric analysis.

Extracted peptides were analyzed on an LTQ Orbitrap Velos mass spectrometer (Thermo Fisher Scientific) and protein identified using Thermo Proteome Discoverer™version 1.4.0 (Thermo Fisher Scientific) and Mascot search algorithm as described previously (Weldearegay et al., 2016).

### 2.4 Adhesion inhibition assay of identified antigens

Recombinant plasmids capable of expressing the genes encoding the proteins corresponding to the antigens identified by mass spectrometry were synthesized by GenScript. The ability of the corresponding AMMY to recognize the recombinant protein expressed from these constructs was tested by Western blotting as described above.

Polyclonal rabbit serum against the recombinant protein was produced by immunizing rabbits intramuscularly with 75 μg protein in 100 μl PBS mixed with equal volumes of Titermax adjuvant on days 0 and 14. Antibody titers were measured by standard indirect ELISA, and cytoadherence inhibition assay was performed as described above. Rabbits were used in accordance with the International Livestock Research Institute Institutional Care and Use Committee guidelines (IACUC Ref No. 2015-19).

### 2.5 Growth inhibition and latex agglutination

All AMMY mAbs were diluted 1:10, sterile filtered and 40 μl of each mAb was added into 2 wells of a 96 well round bottom microplate. Log phase culture of *Mmm* strain Afadé was diluted 1:625 (approximately 2.5 × 10^6^ mycoplasmas/ml) in complete PPLO medium and 160 μl inoculated into each well of the microplate, and incubated at 37 °C for 72 h. PPLO without supplements, anti-*Mmm* strain Afadé rabbit serum and previously published anti-*Mmm* polysaccharide antibodies PK2 (Rurangirwa et al., 2000), 72/27.9.9, 72/16/. 2.14 and 72/18/11.7 (Kiarie et al., 1996) that inhibited growth of *Mmm* were used as controls. The mAbs that inhibited growth were then 10 fold serially diluted to determine the inhibition end point. To confirm whether the mAbs were mycoplasmacidal or mycoplasmastatic, a 50 μl sample was taken from the 72 h culture and inoculated onto PPLO agar, which was then incubated for 5 days at 37 °C and the concentration of the viable cells in the sample determined.

To further understand the mechanism of growth inhibition, a latex agglutination test was performed as previously described (Abdoel and Smits, 2007). Briefly 150 μl of 0.8 μm latex beads were mixed with 2.5 μg of anti-*Mmm* antibodies and the mixture incubated at room temperature for 2 h. The mixture was washed with PBST to remove excess antibody and the beads recovered by centrifugation at 15,000 x g for 3 min, and resuspended in 1 ml of 0.05% sodium azide. Thirty μl of *Mmm* whole cell lysate was placed on a glass slide and thoroughly mixed with 5 μl of the antibody and beads mixture (approximately 75 μg of beads), and then incubated for 10 min on a rocking shaker with gentle agitation.

## 3 Results

### 3.1 Inhibition of cytoadherence

Eighty hybridomas were cloned by limiting dilution from three fusions and 48/80 were positive on ELISA (results not shown).

**Fig. 1:**
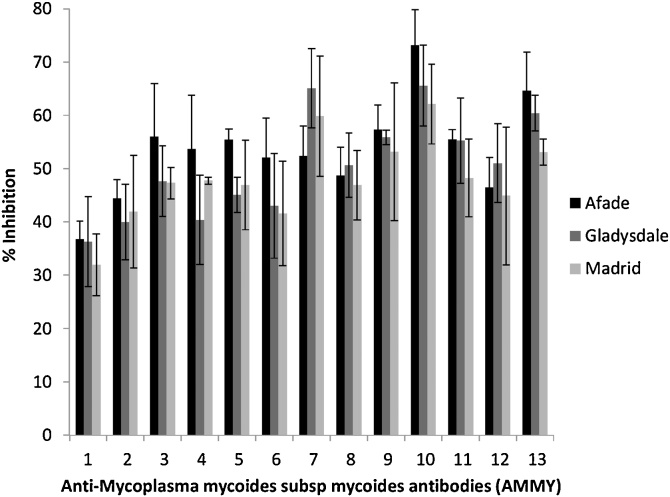
Adhesion inhibition of anti- *Mycoplasma mycoides* subsp *mycoides* (*Mmm*) specific monoclonal antibodies (AMMY). Inhibitory effect of AMMY on the adhesion of three *Mmm* strains to BoLEC at 1:10 dilution. The given adhesion inhibition rates represents the mean value ± standard error from three independent sets of experiments.

Thirteen of the 48 antibodies were able to inhibit adhesionto BoLEC by at least 30% (Fig. S1). The 13 antibodies were renamed AMMY 1–13 and their ability to inhibit adhesion of *Mmm* strain Afadé to BoLEC was tested at varying dilutions (Table 1) and with 3 different strains of *Mmm* (Fig. 1). Mouse pre-immune serum and another mouse IgG mAb served as negative controls. At a dilution of 1:10, the inhibitory effect of the AMMY ranged from 36.73% to 73.17%.

**Table 1:** Percent inhibition of *Mycoplasma mycoides* subspecies *mycoides* adhesion to by anti-*Mycoplasma mycoides* subsp. *mycoides* monoclonal antibodies (AMMY) at various dilutions.

		Antibody dilutions					
		1:1000	1:500	1:250	1:100	1:30	1:10
Percent Inhibition (±SD)	Pre-immun serum	0	0	0	9	9	12
	Mouse Antibody	0	0	0	11	17	18
	AMMY 1	14 (3.4)	26 (16.0)	32 (6.5)	32 (6.8)	33 (7.5)	37 (5.3)
	AMMY 2	13 (6.1)	28 (3.2)	30 (12.2)	33 (10.4)	40 (2.9)	44 (2.2)
	AMMY 3	12 (9.9)	30 (6.1)	36 (1.4)	43 (10.8)	53 (5.7)	56 (2.5)
	AMMY 4	18 (5.1)	41 (6.5)	40 (9.7)	41 (5.7)	43 (5.3)	54 (9.4)
	AMMY 5	17 (2.0)	34 (5.2)	39 (7.1)	41 (5.1)	52 (8.9)	55 (5.0)
	AMMY 6	25 (4.4)	41 (8.3)	43 (12.4)	44 (4.6)	52 (2.4)	52 (10.9)
	AMMY 7	17 (5.7)	38 (4.9)	37 (3.4)	46 (8.2)	50 (9.8)	52 (7.9)
	AMMY 8	21 (5.3)	33 (6.1)	35 (4.6)	40 (4.1)	47 (3.3)	49 (6.3)
	AMMY 9	18 (4.5)	35 (8.4)	39 (9.7)	43 (10.1)	53 (3.8)	57 (5.0)
	AMMY 10	22 (2.8)	40 (10.6)	47 (8.2)	52 (4.1)	69 (2.5)	73 (4.6)
	AMMY 11	14 (5.0)	25 (1.8)	31 (9.1)	35 (6.4)	49 (2.5)	55 (8.5)
	AMMY 12	27 (8.8)	35 (5.7)	36 (13.0)	35 (9.9)	37 (8.8)	46 (4.8)
	AMMY 13	17 (8.4)	30 (7.1)	46 (6.6)	56 (8.3)	62 (3.3)	65 (9.8)

The isotypes of the 13 AMMY were IgG2b (AMMY 1–4 and 11–13), IgG2a (AMMY 5, 7 and 8), IgG1 (AMMY 6 and 9) and IgM (AMMY 10).

Schieck et al. (2016) characterized AMMY 10 as an *Mmm*-specific capsular polysaccharide (CPS) antibody.

### 3.2 Antigen identification and cytoadhesion inhibition

**Fig. 2:**
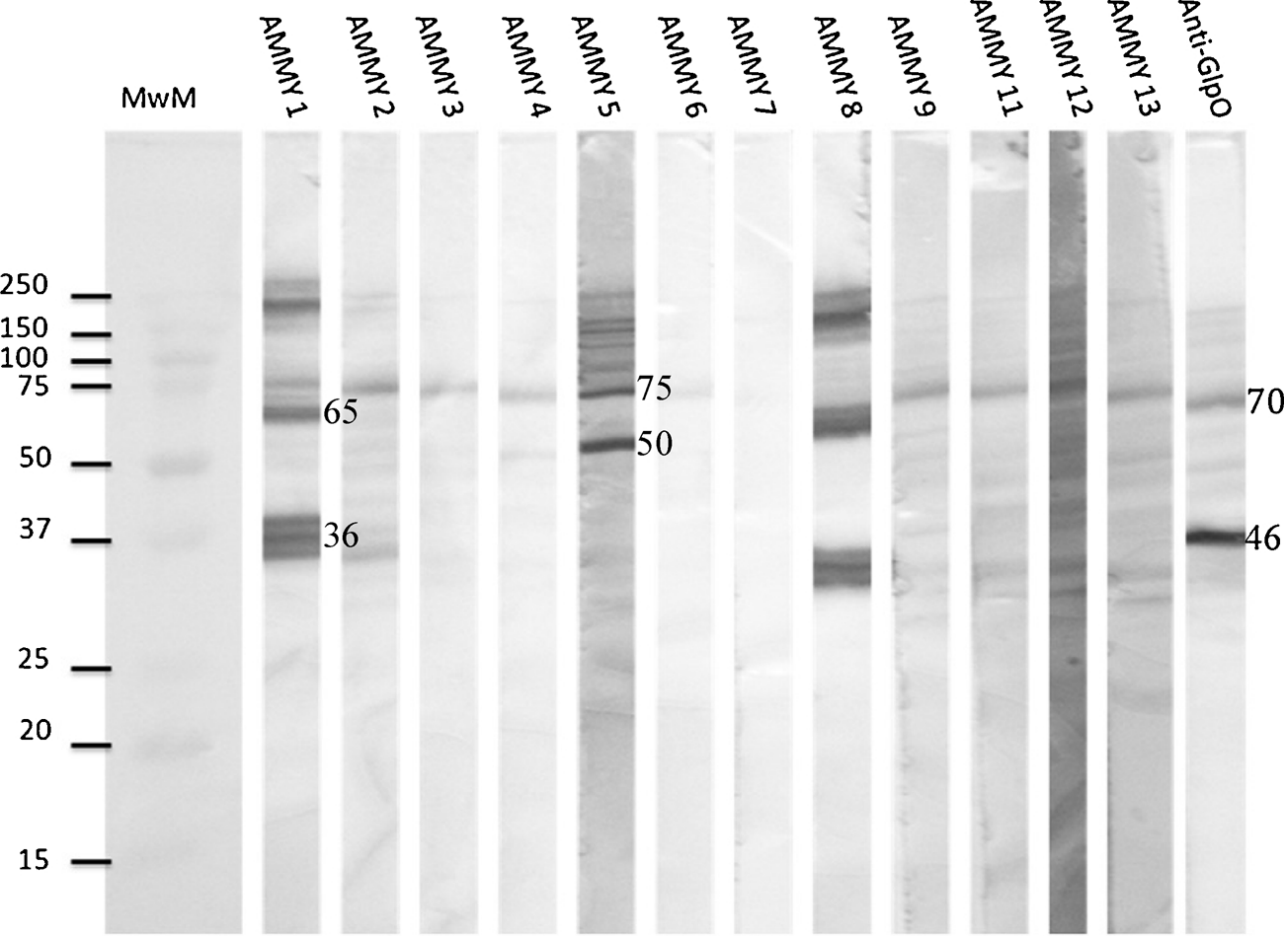
Western blot analysis of anti-*Mycoplasma mycoides* subsp. *mycoides* (AMMY) monoclonal antibodies. *Mycoplasma mycoides* subsp. *mycoides* (*Mmm*) strain Afadé whole cell lysate was transferred onto nitrocellulose membrane and probed with AMMY mAbs. Anti-GlpO was used as a positive control. Membrane stained with *Mmm* specific monoclonal antibodies raised against strain Afadé (1:1000) and HRP conjugated goat anti-mouse (1:2500) (Sigma) and visualized by DAB.

Ten of the residual 12 mAbs were able to recognize *Mmm* proteins on Western blots. AMMY 1 and AMMY 8 recognized the same proteins with molecular weights of 223, 75 and 36 kDa, whereas AMMY 5 recognized two proteins with molecular weights 75 and 50 kDa. The rest of the AMMY antibodies appeared to recognize a protein of approximately 70 kDa (Fig. 2). An anti-GlpO mAb (Mulongo et al., 2013) was used as a positive control. Since the 70 kDa protein was detected in blots probed by most of the AMMY antibodies and the positive control, we concluded that this binding was not specific and the antibodies binding to this protein were not prioritized for further analysis.

**Fig. 3:**
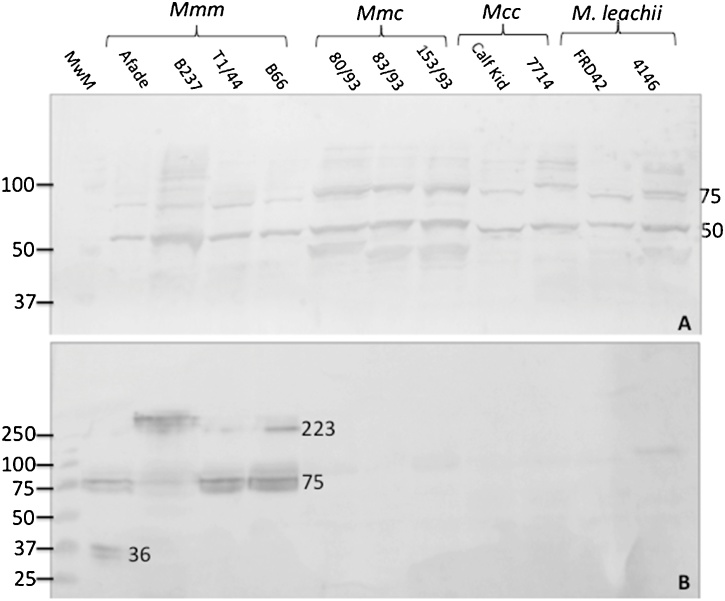
Species specificity of anti-*Mycoplasma mycoides* subsp. *mycoides* (AMMY) monoclonal antibodies. The ability of AMMY monoclonal antibodies to recognize proteins from different subspecies of the *M*. *mycoides* cluster was assessed. (A) AMMY 5 against different subspecies of the *M*. *mycoides* cluster. (B) AMMY 8 against different subspecies of the *M*. *mycoides* cluster. The *M*. *mycoides* subspecies tested include *M*. *mycoides* subsp. *mycoides* (*Mmm*) strains Afadé, B237, T144 and B66; M. *mycoides* subsp. *capri* (*Mmc*) strains 80/93, 83/93 and 153/93; *M*. *capri* subsp. *capripneumonia* (*Mcc*) strains Calf Kid and 7714; *M. leachii* strains FRD42 and 4146. Membrane stained with *Mmm* specific monoclonal antibodies raised against *Mmm* strain Afadé (1:1000) and goat anti-mouse HRP conjugated (1:2500) (Sigma) and visualized by DAB.

The specificity of Western blot positive antibodies for proteins of *Mmm* among members of the closely related *Mycoplasma mycoides* cluster was assessed. AMMY 5 was found to recognize proteins present in all the subspecies tested (Fig. 3A) whereas AMMY 1 and AMMY 8 recognized proteins specific for *Mmm* only (Fig. 3B).

**Fig. 4:**
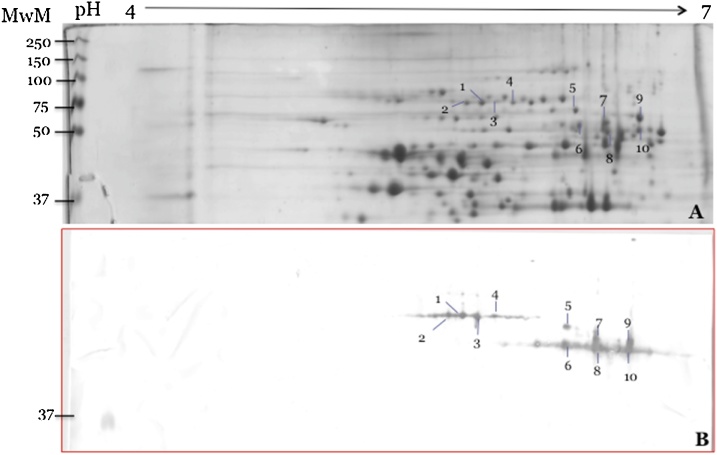
Two-dimensional electrophoresis and immunoblot analysis of *Mycoplasma mycoides* subsp. *mycoides*. Total cell lysate proteins of *Mmm* strain Afadé (1 mg) were separated on pH 4–7 non-linear ImmobilineTM Dry Strips and 12.5% SDS–PAGE. (A) Stained with colloidal Coomassie G250. (B) Further analyzed by immnuo blot. Membrane stained with *Mmm* specific monoclonal antibodies raised against strain Afade (AMMY 5, 1:1000) and goat anti-mouse alkaline phosphatase conjugated (1:2500) and visualized by BNIP/NCIT, and proteins in the molecular mass standard are indicated on the right of the gels.

Using 2-DE and Western blotting, two groups of spots recognized by AMMY 5 (Fig. 4A and B) were detected. The first group (spots 1–4) had an apparent molecular weight of 75 kDa, the second group (spots 5–10) was found at about 50 kDa- both values corresponded well with the major immunoreactive bands detected in 1D gels (Fig. 2). As non-linear strips were used for electrofocusing, the exact pIs of the spots could not be deduced from the spot positions.

Eight spots (numbers 1, 4–10) were successfully analyzed by LC–MS/MS. All eight spots analyzed were identified as containing multiple proteins and the results were sorted according to total peptide scores, sequence coverage, number of unique peptides and frequency of protein detection within the immunoreactive spots (Suppl. Table 1). The most abundant proteins found in each spot are listed in Table 2.

**Table 2:** Most abundant proteins recognized by anti-*Mycoplasma mycoides* subsp. *mycoides* (AMMY 5) monoclonal antibody as identified by LC–MS/MS.

Spot	Accession^a^	Protein name	locus tag^b^	gene name	MW [kDa]	calc. pI
1	D9QWT6	Dihydrolipoyl dehydrogenase	MSC_0268	*lpdA*	64.2	5.72
4	D9QWT5^c^	2-oxo acid dehydrogenase acyltransferase	MSC_0267	*pdhC*	45.8	6.67
5	D9QVZ9	DNA gyrase subunit B	MSC_0006	*gyrB*	71.5	5.94
6	D9QX02	Aspartate--tRNA ligase	MSC_0333	*aspS*	66.6	6.00
7	D9QYF8	glycerate mutase	MSC_0825	*gpmL*	60.1	6.33
8	D9QWS9	Pyruvate kinase	MSC_0261	*pyk*	53.8	6.40
9	D9QWT5^c^	2-oxo acid dehydrogenase acyltransferase	MSC_0267	*pdhC*	45.8	6.67
10	D9QWT5^c^	2-oxo acid dehydrogenase acyltransferase	MSC_0267	*pdhC*	45.8	6.67

a Uniprot accession number.

b Genome sequence *M*. *mycoides* strain PG1 (BX293980).

c Most probable protein to AMMY 5.

The antigen most probably recognized by AMMY 5 was 2-oxo acid dehydrogenase acyltransferase, encoded by the *pdh*C gene (MSC_0267). MSC_0267 was identified as the most abundant protein in three spots, with comparably high numbers of unique peptides. In the additional two spots, MSC_0267 was the second or third most abundant protein (Suppl. Table 1).

**Fig. 5:**
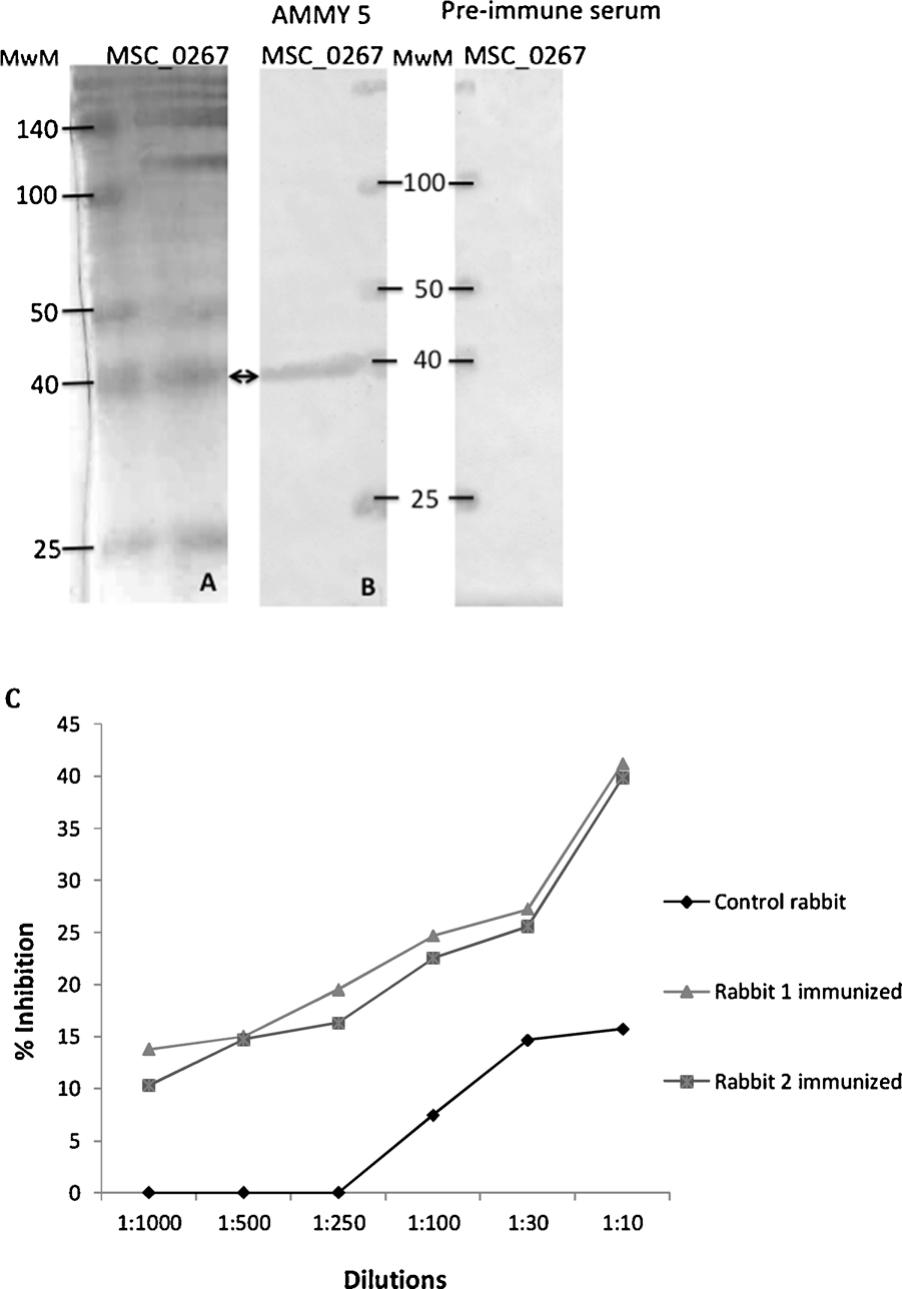
Functional analysis of recombinant MSC-0267. (A) rMSC_0267 was run on a SDS-PAGE gel and stained with Coomassie blue (B) Western blot analysis of rMSC_0267. Membrane was probed with anti-*Mycoplasma mycoides* subsp. *mycoides* (AMMY) 5 monoclonal antibody. Mouse preimmune serum was used as a negative control. Membrane stained with *Mmm* specific monoclonal antibodies raised against strain Afadé (1:1000) and HRP conjugated goat anti-mouse (1:2500) (Sigma) and visualized by DAB. Black arrow, rMSC_0267 band. (C) Adhesion inhibition by polyclonal rabbit serum against 2-oxo acid dehydrogenase acyltransferase (Catalytic domain) (MSC_0267).

AMMY 5 was shown to bind recombinant MSC_0267 on Western blot (Fig. 5B), confirming our identification above. Additionally, polyclonal rabbit serum raised against recombinant MSC_0267 inhibited adherence by 41% (Fig. 5C)

### 3.3 Growth inhibition

**Fig. 6:**
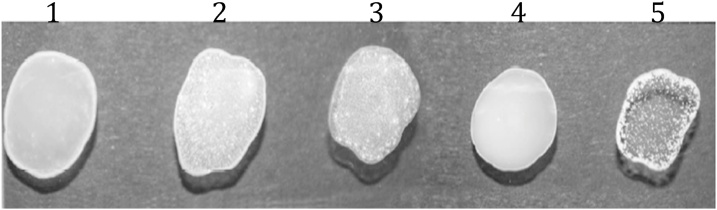
*Mycoplasma mycoides* subsp. *mycoides* (*Mmm*) latex agglutination test. (1) Uncoated latex beads with *Mmm* total cell lysate. (2) Latex beads coated with Anti- *Mmm* rabbit serum plus *Mmm* total cell lysate. (3) Latex beads coated with unpurified Anti- *Mmm* (AMMY 10) plus *Mmm* total cell lysate. (4) Latex beads coated with control mouse IgM plus total cell lysate. (5) Latex beads coated with purified AMMY 10 plus *Mmm* total cell lysate. Agglutination was rated as (−) absent for samples 1 and 4, (+) strong for samples 2 and 3, and (+++) very strong for sample 5.

One out of thirteen mAbs (AMMY 10) was able to inhibit growth (Suppl. Table 2) at a dilution of 1:10,000 (Table 3) and when added to latex beads, agglutination was observed (Fig. 6). Moreover, when the *Mmm* and antibody mixture was inoculation on PPLO agar, growth was inhibited on confirming the mycoplasmacidal effect of AMMY 10.

**Table 3:** *In vitro* growth inhibition of *Mycoplasma mycoides* subsp. *mycoides* (*Mmm*) by anti-*Mmm* 10.

	A	B	C	D	E	F				
	10	10^2^	10^3^	10^4^	10^5^	10^6^	10^7^	10^8^	10^9^	10^10^
Controls	+	–	–	–	+	–				
AMMY 10	+	+	+	+	–	–	–	–	–	–
pK2	+	+	+	–	–	–	–	–	–	–
72/27.9.9	+	–	–	–	–	–	–	–	–	–
72/16.2.14	+	–	–	–	–	–	–	–	–	–
72/18.11.7	+	–	–	–	–	–	–	–	–	–

Experiment performed in PPLO-media with phenol red as a color change indicator. AMMY 10 diluted 1:10. Anti- *Mmm* carbohydrate (pK2) and antibody to surface proteins 72/27/9.9, 72/16/2.14 and 72/18/11.7 that inhibited growth were used as positive controls, also diluted 1:10. Controls includes (A) Plain media; (B) *Mmm* strain Afadé in media without antibody; (C) *Mmm* strain Afadé plus PBS; (D) *Mmm* strain Afadé plus pre-immunization rabbit serum; (E) *Mmm* specific rabbit serum; and (F) *Mmm* plus mouse control antibody. + Inhibited growth; − did not inhibit growth.

## 4 Discussion

Antibodies that inhibit adhesion, whether by binding to the adhesive epitope or by steric hindrance, might detect *Mycoplasma* antigens that are vaccine candidates, as these would induce functional antibodies with a capacity to prevent adhesion and/or growth, and potentially colonization of the lungs. Thirteen AMMYs significantly reduced adherence of *Mmm* to BoLEC by 30–70%. These values were with in range reported in studies on inhibition of adherence of other mycoplasmas. A monoclonal antibody against a 26 kDa *M. bovis* protein was able to inhibit cytoadherence of two *M. bovis* strains by 46% and 70% respectively (Sachse et al., 1993). Similarly, anti-P1 antibody inhibited the adhesion of *M*. *pneumoniae* by 80% (Krause and Baseman, 1983).

AMMY 10, an *Mmm*-specific capsular polysaccharide (CPS) antibody (Schieck et al., 2016), had the greatest capacity to inhibit adhesion, inhibited growth and caused agglutination of *Mmm* lysate when coated with latex beads suggesting that this antibody does not block the adhesin-receptor interaction but rather agglutinates and kills the mycoplasmas hence rendering them unable to adhere. The role of anti-*Mmm* carbohydrate in inhibition of growth has been reported previously (Rurangirwa et al., 2000). AMMY 10 is an IgM antibody and thus is likely to be particularly effective in complement activation, and might contribute greatly to opsonization (Ding et al., 2013). Immunization of cattle with a capsular polysaccharide (CPS) formulated vaccine elicited antibody responses with titers similar to those elicited by the live vaccine and reduced pathology by 57% (Mwirigi et al., 2016). CPS plays a role in the capacity of *Mmm* to persist and disseminate in the infected host (March et al., 2000) further underscoring the potential of *Mmm* carbohydrate as a vaccine candidate.

Ten monoclonal antibodies bound to multiple protein bands on western blots. This could be because they bound to proteins having two or three isoforms with mass and/or charge differences indicating post-translational modification of the proteins (Jores et al., 2009), or because of repetitive sequences in the *Mmm* genome, resulting in proteins/protein coding genes of differing sizes that contain the same epitope (Westberg et al., 2004; Bischof et al., 2006).

The protein identified upon 2D-gel electrophoresis, 2-oxo acid dehydrogenase acyltransferase (Catalytic domain) (MSC_0267), is part of the pyruvate dehydrogenase (PDH) complex, which also contains pyruvate dehydrogenase (lipoamide) alpha chain (MSC_0265), pyruvate dehydrogenase (lipoamide) beta chain (MSC_0266) and dihydrolipoamide dehydrogenase (MSC_0268). In *Mmm*, the pyruvate dehydrogenase complex is an immunogenic protein located in the membrane (Krasteva et al., 2014) that is recognized by sera from experimentally infected cattle (Jores et al., 2009). The catalytic domain of the complex catalyzes the overall conversion of alpha-keto acids to acyl-CoA and carbon dioxide, an irreversible step in the utilization of carbohydrate (Perham, 1991), suggesting that MSC_0267 plays a crucial role in the survival of *Mmm* in the host. The pyruvate dehydrogenase complex has been shown to mediate adhesion of *M*. *pneumoniae* to fibronectin (Dallo et al., 2002) and plasminogen (Thomas et al., 2013; Grundel et al., 2015), a very common component of eukaryotic cell surfaces and basement membranes. Antibodies to PDH were able to inhibit adherence of *M*. *pneumoniae* to fibronectin, but had little or no effect on adherence to human epithelial cells, leading the investigators in this study to speculate that the interaction of these proteins with host components is important in later stages of colonization. These include protection against the host’s immune response (Madureira et al., 2007; Feng et al., 2009;), adherence to deeper layers of the epithelium with higher concentrations of extracellular matrix (ECM) molecules (Yavlovich et al., 2004; Grundel et al., 2016), and access of the bacteria to more favorable nutrient conditions. On the other hand, interaction with plasminogen may also increase bacterial adhesiveness. Plasminogen binds to bacterial cell surface receptors as well as to integrin molecules on eukaryotic cells and can thus also enhance bacterial adherence to host epithelia through a bridging mechanisms (Pluskota et al., 2004; Siemens et al., 2011; Sanderson-Smith et al., 2012). The process enhances surface-bound proteolytic activity that can enhance bacterial survival or spread within the host (Lähteenmäki et al., 2005). PDH is an example of a ‘moonlighting protein’. The proteins have more than one function, playing a role in essential cellular processes (canonical functions), and also an auxiliary role, such as binding to host cells and the ECM (moonlighting functions) (Kainulainen and Korhonen, 2014). In our study, polyclonal rabbit anti-serum raised against MSC_0267 inhibited adhesion of *Mmm* to BoLEC by 41%. Further investigations may help to provide a more comprehensive picture of the mechanisms involved in the overall success of *Mmm* in colonizing the host respiratory tract. The studies described here suggest that MSC_0267, a component of the pyruvate dehydrogenase complex, may be a structural component of the adherence system.

## 5 Conclusion

A panel of *Mmm*-specific monoclonal antibodies was developed and their ability to inhibit *Mmm* cyto-adherence to BoLEC and inhibit growth was assessed. The data suggest that the mechanism of *Mmm* adherence to BoLEC is complex, involving a variety of mycoplasmal antigens, as evidenced by the inhibitory effect of various antibodies. There were similarities to the mechanisms involved in the adherence of *M. pneumoniae* and *M. bovis*. Our approach allowed us to identify an anti-*Mmm pdhC* specific mAb (AMMY 5), which bound to an antigen that appears to play a vital role in cellular processes. We further described the adhesion and growth inhibitory capacity of AMMY 10, a previously characterized anti- *Mmm* CPS mAb. Antigens recognized by these antibodies could be vaccine candidate(s) that might induce antibodies that inhibit adhesion, growth and other important biological functions, and prevalent colonization of the lung by *Mmm*.

## Competing interests

The authors declare that they have no competing interests.
