# Supplementary material for: Identification of targets of monoclonal antibodies that inhibit adhesion and growth in Mycoplasma mycoides subspecies mycoides
Source: Vet Immunol Immunopathol. 2018 Oct;204:11–8. doi: 10.1016/j.vetimm.2018.09.002 (PMC6215757; doi:10.1016/j.vetimm.2018.09.002)
Supplement: Supplementary file 2 [file mmc2.docx]

**S2 table 2: *In vitro* growth inhibition of Anti-*Mycoplasma* *mycoides* subsp. *mycoides* (AMMY) monoclonal antibodies.**

|  | 1 | 2 | 3 | 4 | 5 | 6 | 7 | 8 | 9 | 10 | 11 | 12 |
| --- | --- | --- | --- | --- | --- | --- | --- | --- | --- | --- | --- | --- |
| A | - | - | - | - | + | + | + | + |  |  |  |  |
| B | - | - | - | - | - | - | - | - | - | - | - | - |
| C | - | - | - | - | - | - | + | + | - | - | - | - |
| D | - | - |  |  |  |  |  |  |  |  |  |  |

Experiment performed in duplicated in PPLO-media with phenol red as a color change indicator. Controls included A1, 2: Phosphate buffered saline, A3, 4: Pre-immune mouse serum, A5, 6: Anti-*Mycoplasma mycoides* subsp. *mycoides* (*Mmm*) mouse serum, A7, 8: Anti- *Mmm* carbohydrate (pK2). From B1-D2, the 13 AMMY’s sequentially were tested. C7, 8 shows AMMY10 that inhibited growth. + Inhibited growth; - did not inhibit growth
