# Supplementary material for: Identification of targets of monoclonal antibodies that inhibit adhesion and growth in Mycoplasma mycoides subspecies mycoides
Source: Vet Immunol Immunopathol. 2018 Oct;204:11–8. doi: 10.1016/j.vetimm.2018.09.002 (PMC6215757; doi:10.1016/j.vetimm.2018.09.002)
Supplement: Supplementary file 3 [file mmc3.docx]

**S1 fig: Adhesion inhibition of anti- *Mycoplasma mycoides* subsp *mycoides* (AMMY) monoclonal antibodies (mAbs).** Only mAbs that inhibited *Mycoplasma mycoides* subsp *mycoides* strain Afade adhesion to bovine lung epithelial cells by at least 30% were considered for furthur analyses.
